# Editorial: Personalized medicine in CKD patients

**DOI:** 10.3389/fneph.2023.1270382

**Published:** 2023-08-16

**Authors:** Michele F. Eisenga, Gert Mayer, Markus Pirklbauer, Michele Provenzano

**Affiliations:** ^1^ Division of Nephrology, Department of Internal Medicine, University Medical Center Groningen, University of Groningen, Groningen, Netherlands; ^2^ Department of Internal Medicine IV - Nephrology and Hypertension, Medical University Innsbruck, Innsbruck, Austria; ^3^ Nephrology, Dialysis and Renal Transplant Unit, Scientific Institute for Research, Hospitalization and Healthcare-Azienda Ospedaliero-Universitaria di Bologna, Alma Mater Studiorum University of Bologna, Bologna, Italy

**Keywords:** personalized medicine, nutrition, parathormone, statin, residual renal function (RRF)

Chronic kidney disease (CKD) is a clinical condition associated with an increased risk for cardiovascular (CV) events, all-cause mortality, and end-stage kidney disease (ESKD). Moreover, its incidence and prevalence are rising dramatically, having nearly doubled in the past two decades. A critical issue in CKD patients is their residual risk, namely the presence of modifiable risk factors over time despite treatment with agents aimed at “normalizing” such risk factors. One example is represented by albuminuria. It has been shown that >60% of CKD patients have mild-to-severe albuminuria despite optimal treatment with inhibitors of the renin-angiotensin system. This can be explained by the individual variability in response to treatment. Other than treatment response, a large variability exists in developing ESKD or CV endpoints that are not entirely captured by baseline levels of kidney function (eGFR) or albuminuria. Further effort is needed to identify patients likely to develop a cardio-renal event (prognosis) and those more likely to respond to a specific treatment (prediction). Hence, precision medicine, taking into account personal, social, and environmental factors besides biomarkers and genotyping, is needed. This Research Topic, “*Personalized medicine in CKD patients*”, focuses on the role of personalized medicine in CKD in terms of prognosis and prediction. In this edition, four articles were gathered that investigated forefront topics related to the topic. Chen and Pongpirul highlight in their review the future of medicine in which mobile apps, wearable devices, and artificial intelligence can lead to individualized self-management to lower the risk of CKD or prevent CKD progression. An essential shift towards less CKD development and progression can be acquired by following proper nutritional and lifestyle recommendations. A personalized approach is crucial as lifestyle modifications differ, for example, by comorbidities. As an illustration, those with cardiovascular disease (CVD) should increase their aerobic capacity, whereas those with chronic obstructive pulmonary disease (COPD) should receive high dietary fiber intake and engage in moderate-intensity exercise (1). It is clear that with current technological advancements, these lifestyle modifications could be better implemented and maintained, but greater international engagement is needed to transform current CKD care. In another study, Kim et al. investigate the importance of statins in CKD patients, including hemodialysis (HD) patients. CVD is the most common cause of death in CKD patients and the likelihood of cardiovascular mortality increases with decreasing eGFR (2). Dyslipidemia is a major risk factor for CVD and is increased in CKD patients (3). Hence, it would be logical to prescribe all CKD patients a statin, but contradictory results have previously been found, mainly in HD patients. Kim et al. now show in two extensive observational cohort studies, including 44,431 and 64,165 CKD patients, that statin use is associated with lower cardiovascular and all-cause mortality across various CKD stages. In *post hoc* analyses, the authors also show a beneficial effect in HD patients. The latter effect contrasts with two well-known randomized controlled trials (i.e., 4D and AURORA), which failed to show a protective effect for atorvastatin in diabetic HD patients or rosuvastatin in HD patients aged 50-80 years, respectively (4, 5). The current study confirms the importance of statin use in terms of mortality risk in CKD patients, but together with other recent observational studies (6, 7) questions whether we might need to reconsider or at least thoroughly re-investigate statin use in HD patients. Also, in this Research Topic, the topic of mathematical models is thoroughly approached. Mathematical models are extremely useful for studying complex physiological processes and, thus, have become essential tools for personalized medicine over the last decades. In a current review, Schappacher-Tilp et al. discuss different approaches to model parathyroid gland (PTG) biology in the context of ESKD. Altered PTG biology, such as secondary hyperparathyroidism, is a key mediator of CKD-related mineral bone disorders (CKD-MBD) and is associated with increased cardiovascular morbidity and mortality. The article elegantly discusses how mathematical models of PTG biology can help better predict parathyroid hormone (PTH) dynamics individually, thus allowing for better prognosis and personalized treatment approaches. The impact of different model structures on their clinical utility in HD patients is discussed. Simpler models are of limited clinical use as they only predict the immediate PTH response to intradialytic serum calcium changes. More sophisticated models, however, allow us to accurately predict short- and long-term PTH changes over several months by utilizing readily available clinical parameters. These complex physiology-based PTG models are personalized by estimating key model parameters based on clinical data, such as longitudinal calcium and phosphate levels, PTH history, dialysis vintage, and information about vitamin D therapy. Finally, this Research Topic presents a review by Steinwandel et al. in which the authors investigate the decline of residual renal function (RRF) during the first year of HD. RRF refers to the kidney’s ability to produce urine and excrete waste products despite ongoing CKD. Preserving RRF is crucial in HD patients, with studies showing positive effects over time on comorbidity, health-related quality of life (HRQoL), and cardiovascular disease. The article presents a meticulous and insightful literature review, highlighting the significance of preserving RRF in HD patients. Preserved RRF can lead to a better fluid balance, reduced intradialytic complications, and less severe ventricular hypertrophy, ultimately improving the overall well-being and HRQoL of patients. While RRF preservation offers promising benefits for HD patients, addressing several challenges is crucial. Striking a delicate balance between preserving RRF and preventing fluid overload is essential. Standardizing RRF assessment methods is necessary to optimize clinical practice. Nonetheless, the potential advantages of RRF preservation, including reduced hospitalizations and improved patient outcomes, outweigh these limitations, emphasizing the importance of patient-centered care. Understanding the trajectory of RRF and its impact on clinical outcomes will inform evidence-based practices and personalized therapy.

In conclusion, this Research Topic, “*Personalized medicine in CKD patients*”, presents interesting new perspectives and highlights the importance of personalized medicine, ranging from new technological devices aiding nutritional management to sophisticated mathematical models of PTG biology. Personalized medicine is a relatively new field that is only starting to open up, but it will undoubtedly become the future of CKD care.

## Author contributions

ME: Conceptualization, Writing – original draft, Writing – review & editing. GM: Writing – review & editing. MPi: Writing – original draft, Writing – review & editing. MPr: Writing – original draft, Writing – review & editing.
